# Investigating the Advantages of Ultrasonic-assisted Welding Technique Applied in Underwater Wet Welding by in-situ X-ray Imaging Method

**DOI:** 10.3390/ma13061442

**Published:** 2020-03-21

**Authors:** Hao Chen, Ning Guo, Kexin Xu, Cheng Liu, Guodong Wang

**Affiliations:** 1 State Key Laboratory of Advanced Welding and Joining, Harbin Institute of Technology, Harbin 150001, China; chh523@126.com (H.C.); wanggd@mail.neu.edu.cn (G.W.); 2 Shandong Provincial Key Laboratory of Special Welding Technology, Harbin Institute of Technology at Weihai, Weihai 264209, China; 19S155065@stu.hit.edu.cn (K.X.); liucheng@hit.edu.cn (C.L.); 3 Shandong Institute of Shipbuilding Technology, Weihai 264209, China

**Keywords:** underwater wet welding, FCAW, ultrasonic-assisted, microstructure refinement, in-situ X-ray imaging

## Abstract

In this study, the effects of ultrasonic on melt pool dynamic, microstructure, and properties of underwater wet flux-cored arc welding (FCAW) joints were investigated. Ultrasonic vibration enhanced melt flow and weld pool oscillation. Grain fragmentation caused by cavitation changed microstructure morphology and decreased microstructure size. The proportion of polygonal ferrite (PF) reduced or even disappeared. The width of grain boundary ferrite (GBF) decreased from 34 to 10 μm, and the hardness increased from 204 to 276 HV. The tensile strength of the joint increased from 545 to 610 MPa, and the impact toughness increased from 65 to 71 J/mm^2^ due to the microstructure refinement at the optimum ultrasonic power.

## 1. Introduction

Underwater wet welding and repair have been widely used in the field of marine constructions, such as nuclear power stations, offshore platform, and gas pipelines [1,2]. It also can be used in the emergency repair of submarine and warship in wartime due to its outstanding operability. However, directly contacting with surrounding water will bring some problems, which deteriorate welding stability and quality [3]. The obvious question is that the heat loss caused by the water environment is much more than that of welding in the air [4]. The rapid cooling rate of molten metal will induce the generation of brittle martensite in the steel welded joints, especially in the heat-affected zone (HAZ) [5,6].

In order to reduce the cooling rate and maintain arc burning in water, the higher welding current and arc voltage are usually used in the welding process [7]. But high heat input easily leads to coarse grains and coarse microstructure in the weld metal, such as coursing proeutectoid ferrite [8,9]. Zhang et al. investigated the application of the real-time induction heating method in the underwater wet welding process [10]. The results showed that the cooling rate of the joints in underwater wet welding was reduced by introducing the induction heating during the welding process. Tomków et al. studied the effect of temper bead welding technique on the weldability of the S460N steel during the underwater wet welding [11,12]. They improved the microstructure of weld metal and decreased the number of cold cracks in the HAZ by using a temper bead welding technique. Guo et al. found that the adding of Ni powders in the electrodes could help decrease the amount of coarse pro-eutectoid ferrite in the weld metal [13]. In addition, it also could refine the microstructure and enhance the mechanical properties of welded joints. Zhang et al. observed that the average grain size was reduced by 22.5% by employing workpiece vibration at a lower frequency [14]. They believed that workpiece vibration could effectively refine the grain size. Because the introduction of the bending stress can break the dendrite arms and promote the production of more nuclei, Sun et al. created an acoustic field between the workpiece and the ultrasonic radiator by introducing high-frequency ultrasonic wave [15]. Their study results showed that the arc stability was improved, and the amount of martensite (M) and upper bainite (BU) in weld metal was decreased. Yuan et al. confirmed that ultrasonic vibration could significantly change the microstructure of weld metal by dipping an ultrasonic probe in the weld pool to directly introduce the ultrasonic energy [16]. Chen et al. propagated ultrasound into the weld pool through the base material by pressing the ultrasonic horn onto the surface of the base material [17]. The result demonstrated that the grain of the tungsten inert gas (TIG) weld of pure aluminum was periodically broken, caused by a periodic ultrasound. Wang et al. found that the application of ultrasonic waves could reduce the fluctuations of the larger arc voltage signal and smaller arc voltage signal [18]. Krajewski et al. researched the ultrasonic-vibration assisted arc-welding of aluminum alloys using the melt inert-gas welding (MIG) and the tungsten inert gas welding (TIG) methods [19]. They found that in the TIG welding, the weld width and weld penetration depth increased, whereas, after MIG welding, the width was narrower. Besides, ultrasonic-assisted processing is used widely in the casting field due to its degassing effect. Some reports have suggested that the ultrasonic could significantly suppress the formation of pores in the molten metal [20,21,22]. 

At present, the mainstream view is that ultrasonic energy results in acoustic pressure and acoustic streaming, which could affect the melt flow and the solidification of the weld pool. However, it is difficult to directly observe the morphological change of the weld pool affected by ultrasonic in the underwater environment. So, the numerical analysis method is often used to investigate the acoustic pressure changes and fluid flow of the melt pool [23,24,25].

Due to the short wavelength and strong penetrability, X-ray could be selected as a light source to image the physical phenomena inside the visually opaque materials. Leung et al. investigated defect formation and molten pool dynamics in laser additive manufacturing by in-situ X-ray imaging [26]. Cunningham et al. revealed the keyhole threshold during laser melting using a high-speed X-ray imaging method [27]. In addition, an X-ray imaging method also could overcome the reflection and refraction in the water. So, it could be selected as the light source to observe the melt flow during underwater wet ultrasonic-assisted flux-cored arc welding (UAFCAW). 

In this research, the influence of ultrasonic power on melt flow in the weld pool was observed. The influences of the ultrasonic power on the microstructure and properties of underwater wet welding joints were investigated. The mechanism of microstructure refinement induced by ultrasonic vibration was discussed.

## 2. Materials and Methods

**Figure 1:**
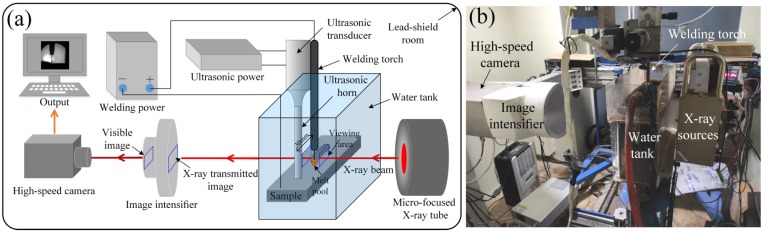
(**a**) Schematic diagram of the experimental setup. (**b**) The experimental platform in a lead-shield room.

Figure 1 shows a schematic of UAFCAW and an in-situ X-ray imaging system. The welding system consisted of an ultrasonic system (KCH-1228), welding power source (DIGI@WAVE500, SAF-FRO, France), moveable platform. The ultrasonic system consisted of ultrasonic power source, ultrasonic transducer, and ultrasonic horn, which was made in Kare Sonic Power Co., LTD, (Weihai, China). All of these were placed in a lead-shield room to protect experimenter from radiation damage from X-ray. The welding was carried out in a water tank driven by a moveable platform using direct current electrode positive (DCEP). The ultrasonic transducer converted electrical energy into ultrasonic vibrations. The ultrasonic vibration was increased by the ultrasonic horn. Then, the horn transferred the ultrasonic to the surface of the sample. In this experiment, the ultrasonic vibration was 27 kHz. During the welding process, the ultrasonic horn was fixed at a constant distance (30 mm) from the welding torch to make sure that it did not melt due to the extreme arc heat.

**Figure 2:**
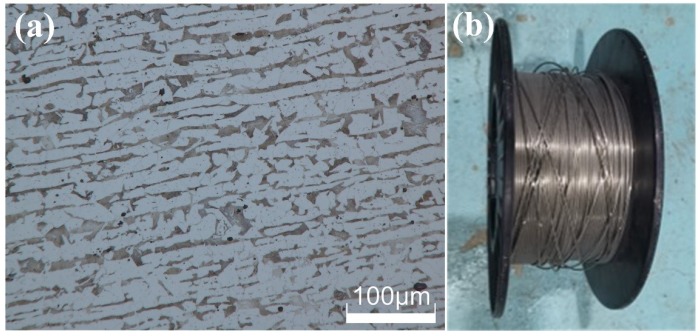
(**a**) Microstructure of EH40 base metal. (**b**) A coil of finished welding wire.

The E40 marine steel with a thickness of 12 mm was selected as base materials in this study. The welding material was a specially developed tubular self-shielded rutile type flux-cored wire with a 1.6 mm diameter. In order to improve the mechanical property, some metal powders, such as Mn and Ni, were added in the flux-core. The low carbon H08A steel strip was the sheath material for the welding wire. The chemical composition of E40 and H08A steel is listed in Table 1. The finished welding wire and the typical microstructure of E40 steel are shown in Figure 2. The microstructure of E40 steel consisted of fine granular ferrite and pearlite. The specific parameters were as follows, welding voltage 28 V, wire feed speed 3.5 m/min, welding speed 120 mm/min, wire extension 15 mm, water depth 0.5 m. The amplitude of ultrasonic vibration was determined by the ultrasonic output power. In order to study the influence of vibration intensity on the welding process, five different ultrasonic output powers of 20%, 40%, 60%, 80%, and 100% of the maximum power (1200 W) were used. Besides, as a comparison, the conventional wet welding without ultrasonic was carried out by the same experimental parameters.

The observations of melt flow and weld pool oscillation were achieved by the in-situ imaging system, as illustrated in Figure 1a. The ultrasonic horn was fixed, and the workpiece and water tank moved along a designed linear path. The X-ray high-speed camera system (CR series, Optronis, Kehl, Germany) was used to collect images of the weld pool during the welding process, as shown in Figure 1b. These images were converted from X-ray transmitted images by the image intensifier. The high-speed images with 1000 fps were captured, and the images, including melt flow, gas evolution, and droplet transfer process, were extracted and analyzed in this research. 

**Figure 3:**
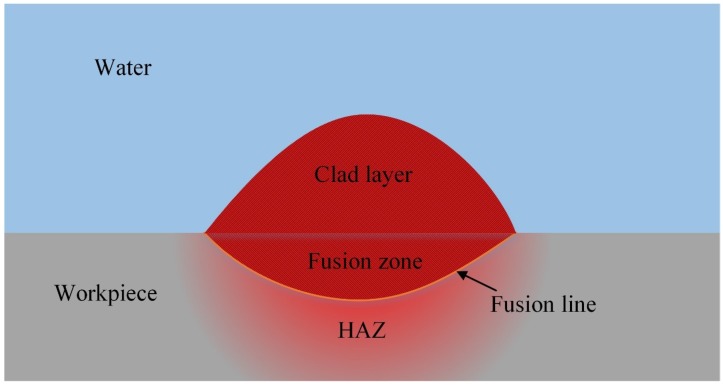
The schematic view of the welded bead geometry characteristic.

To study the effect of ultrasonic on the welded bead geometry, the penetration depth, the area of the fusion zone, and the clad layer were measured, as shown in Figure 3.

The value of weld dilution rate “D” was calculated by the following formula [28]:

(1) $$D=\frac{A_{FZ}}{A_{FZ}+A_{CL}}\times 100\%$$

where A_FZ_ is the cross-section area of the fusion zone, and the A_CL_ is the cross-section area of the clad layer.

**Figure 4:**
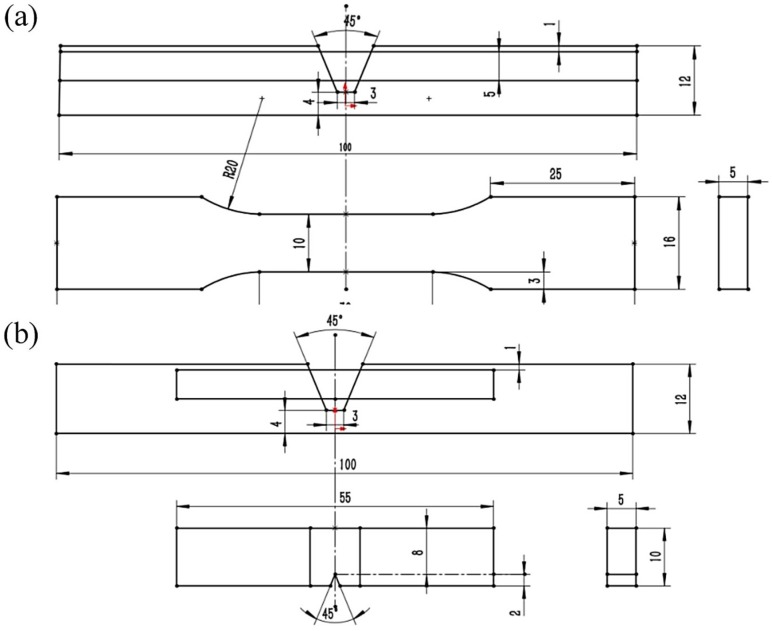
Schematic diagrams of dimensions and extracted locations of (**a**) tensile test samples and (**b**) impact test samples (all dimensions are in mm).

The weld metal was etched with a 4% (vol %) nitric acid ethanol solution. An optical digital microscope (GX51, Olympus, Japan) was used to observe the microstructures. Transverse tensile tests were conducted using a mechanical property testing machine (5967, Instron, Boston, MN, USA) at a pull speed of 2 mm/min. Charpy V-notch impact tests were experimented at room temperature to evaluate the toughness of weld metal. The dimensions and extracted locations of the specimens are displayed in Figure 4. For every arc parameter condition, five specimens were examined in the mechanical properties testing. The Vickers microhardness was measured along the line across the weld metal on the cross-section with a load of 2.942 N for 10 s via an HV-1000DT hardness tester. The observations of fracture surfaces, after the tensile test and Charpy impact test, were completed using a scanning electron microscope (MERLIN Compact, Carl Zeiss, Oberkochen, Germany). 

## 3. Results and Discussion

### 3.1. Weld Geometry

**Figure 5:**
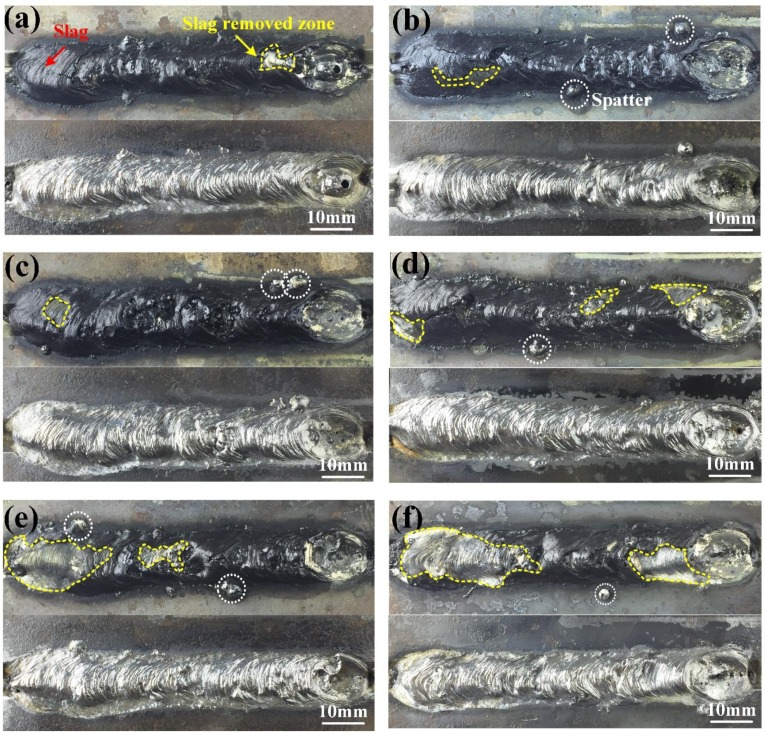
Weld appearances before and after the deslagging at different conditions: (**a**) without ultrasonic; (**b**–**f**) ultrasonic output power were 20%, 40%, 60%, 80%, and 100% of the maximum power (1200 W), respectively.

In this section, the effect of the ultrasonic output power on the weld geometry was investigated. Figure 5 shows the weld appearances before and after the deslagging welded at different ultrasonic output power. Figure 5a shows an acceptable surface appearance obtained without ultrasonic. Only a small portion of the slag was removed automatically after the welding. This result showed that the weld was well covered and protected by the slag. However, the slight distortion and roughness surface could be observed on the weld after the deslagging. Figure 5b–f show the weld bead appearances obtained at 20%, 40%, 60%, 80%, and 100% of the maximum ultrasonic output power, respectively. The application of ultrasonic vibration on the workpiece surface resulted in the appearance of some new phenomenon. The first was the production of the welding spatters. These spatters with a diameter of 3–4 mm were distributed randomly on both sides of the weld bead. The formation process of these spatters could be captured by the imaging system. According to these X-ray images, the formation mechanism of spatters has been revealed in Section 3.2. The amount of welding spatters was increased with the increase of the ultrasonic output power. It was worth noting that these spatters could not be firmly welded with the substrate surface due to the rapid cooling caused by water. Most of these spatters could be easily removed. Second, the vibration caused by ultrasonic improved the melt flow in the weld pool. The defects, such as irregularity of ripples on the weld surface, could be decreased. As shown in Figure 5d, a good weld appearance was obtained as the ultrasonic output power was 60%. The weld was smooth, and there were no obvious defects on the weld surface. Third, the larger portion of the slag was removed induced by the ultrasonic vibration during the welding process. As shown in Figure 5e,f, a large area of slag was removed, which was not conducive to the protection from the impact of water. So, the weld appearance might become worse, and the microstructure and properties of weld would be affected. 

**Figure 6:**
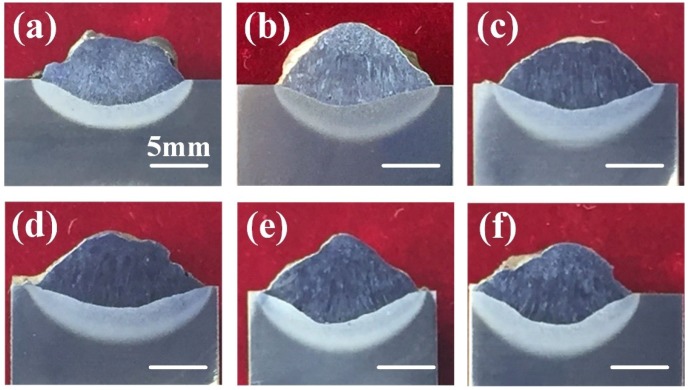
Cross-sections of welded joints obtained at different conditions: (**a**) without ultrasonic; (**b**–**f**) ultrasonic output power was 20%, 40%, 60%, 80%, and 100% of the maximum power (1200 W), respectively.

Cross-sections of welded joints obtained at different ultrasonic output power are illustrated in Figure 6. Defect-free welds could be obtained at different ultrasonic power. It could be found that when the ultrasonic power was relatively low, there was no obvious increase in weld penetration depth compared to that of without ultrasonic, as shown in Figure 6a–d. With ultrasonic power increased to 80% and 100%, the weld penetration depth showed a significant increase, as shown in Figure 6e,f.

**Figure 7:**
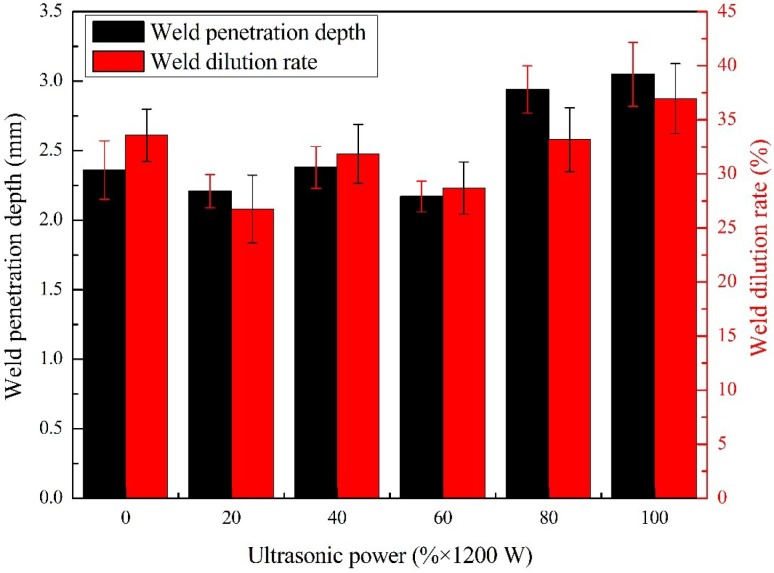
The effect of ultrasonic output power on weld penetration depth and weld dilution rate.

The weld penetration depth and dilution rate were measured and calculated, as given in Figure 7. The weld dilution rate had the same trend of variability compared with that of weld penetration depth.

Some researchers have studied the influence of water on the weld penetration in the wet welding process. For instance, Zhao et al. studied the melt pool behavior of the underwater wet welding process using numerical simulation methods [4]. They pointed out that compared to conventional flux-cored arc welding (FCAW) in the air, there existed a considerable vortex flow dominated by the Marangoni force in the longitudinal section of the melt pool, which transferred a lot of heat to the bottom of the melt pool and resulted in deeper penetration. In this study, the violent fluid flow caused by acoustic streaming increased the heat transfer in the melt pool and accelerated the melting of base metal. In addition, the generation of many cavitation bubbles caused by ultrasonic at the bottom of the melt pool was also one of the reasons that deepened the weld penetration. Many previous types of research have confirmed that the collapse of the ultrasonic cavitation bubble would damage the substrate and form erosion pits [29,30,31]. As shown in the weld transverse image of Figure 6e,f, the fusion lines were not smooth, which was different from the weld obtained by conventional welding or ultrasonic-assisted welding method at low output power. Obviously, the cavitation erosion resulted in a deeper weld penetration and the rough interface between the substrate and deposited metal. This result meant that ultrasonic cavitation promoted more substrate metals to melt into the deposited metal.

### 3.2. Droplet Transfer and Melt Flow

**Figure 8:**
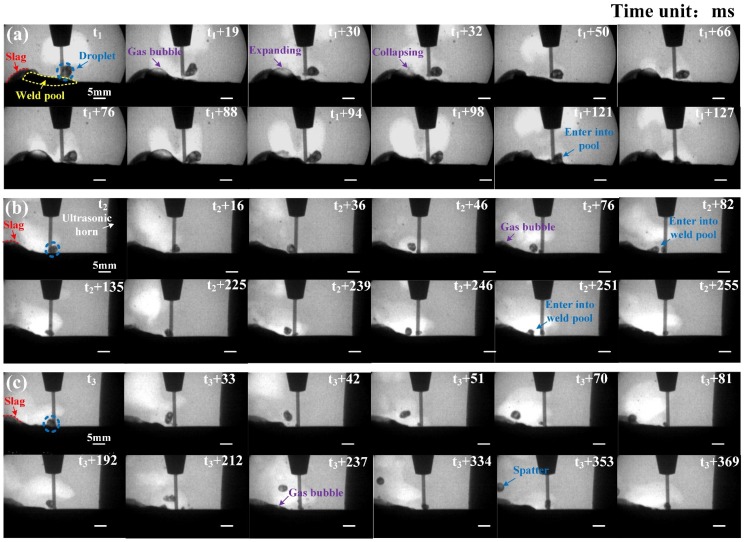
X-ray images of melt flow and droplet transfer process obtained at different conditions: (**a**) without ultrasonic; (**b**,**c**) ultrasonic output power was 40% and 80% of the maximum power (1200 W), respectively.

Figure 8 shows the X-ray images of weld pool dynamics and the droplet transfer process (see details in Supplementary Movie 1–3). As shown in Figure 8a, the gas dissolved in the melt pool formed a gas bubble and expanded in the conventional wet welding. When the volume of the gas bubble was larger enough, this gas bubble would collapse and release the gas into water. The molten droplet showed a large size, and its diameter was about 4–5 mm. The slag covered on the weld was marked by the red arrow. It could be found that it was tightly covered on the weld until the weld pool was solidified, which was consistent with the weld appearance shown in Figure 5a. When the ultrasonic output power was 40% of maximum power, as shown in Figure 8b, the most obvious change was that the gas didn’t escape from the weld pool in the form of a large bubble. Instead, the gas was precipitated and released through forming several smaller gas bubbles at a higher frequency. The melt flow and weld pool oscillation enhanced by the ultrasonic acoustic streaming effect hampered the formation of a large gas bubble. In addition, the droplet diameter decreased to about 2–3 mm, and the droplet transfer showed a shorter cycle time. 

**Figure 9:**
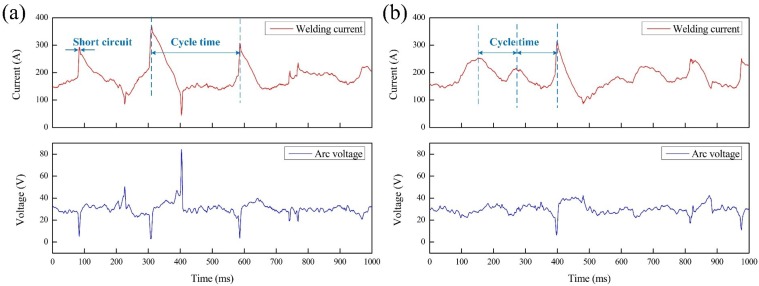
Typical electrical signal waveforms of welding process: (**a**) conventional wet flux-cored arc welding (FCAW), (**b**) ultrasonic-assisted flux-cored arc welding (UAFCAW).

Due to the lower arc voltage, once the droplet detached from the wire, it was followed by a transient short circuit behavior. This phenomenon showed the transient contact between the wire and the weld pool in the images. It also could be characterized by a sudden increase in the welding current. The waveforms diagrams of arc voltage and welding current in two welding processes are displayed in Figure 9. Compared with the conventional wet FCAW without ultrasonic, the time between two current peaks was shorter during the UAFCAW process, as shown in Figure 9b. This result also confirmed that droplet transfer with a shorter cycle time occurred due to the influence of ultrasonic vibration.

Furthermore, it was easier for the droplet to fall onto the side of the weld pool during the droplet transfer process due to the oscillation of water on the other side caused by the ultrasonic horn. As the ultrasonic output power increased to 80% of maximum power, the stronger ultrasonic energy induced a more intense water wave. In this case, the droplet was easier to deviate from the moving track and even became a “droplet repelled spatter”, as shown in Figure 8c. It also could be found that the profile curve of slag was changing constantly. This result indicated that the slag covered on the weld was broken because of the enhanced oscillation of the weld pool during the welding process.

### 3.3. Microstructures and Microhardness

**Figure 10:**
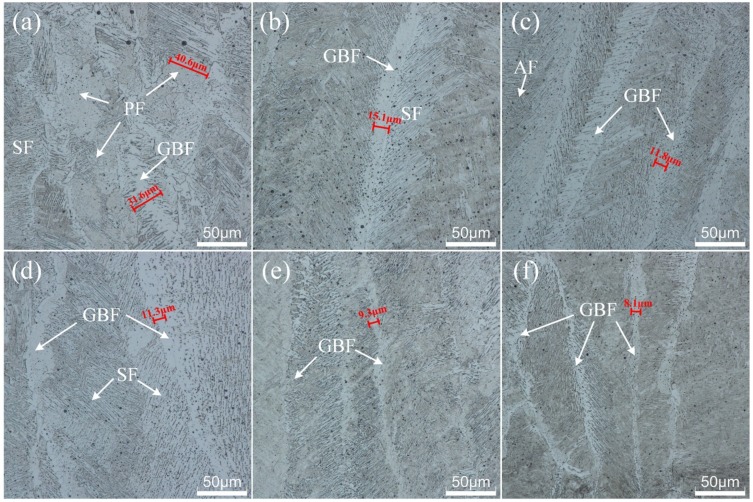
The microstructures of joints in the deposited metal welded at different ultrasonic power: (**a**) without ultrasonic, (**b**) 20%, (**c**) 40%, (**d**) 60%, (**e**) 80%, and (**f**) 100%.

Ultrasonic also had some significant effects on the microstructure of deposited metal. Figure 10 shows the deposited metal microstructures obtained under different ultrasonic output power during the underwater wet welding process. In general, the microstructures in the deposited metal consist of four types of ferrite: polygonal ferrite (PF), grain boundary ferrite (GBF), side plate ferrite (SPF), and acicular ferrite (AF) [9]. As shown in Figure 10a, when welding was without ultrasonic-assisted, the content of PF showed a high proportion and a larger grain size of about 40.6 μm. In addition, the width of GBF distributed around the PF was approximately 31.6 μm. When the ultrasonic power increased to 20% of the maximum output power, the number of PF reduced significantly or even disappeared. The microstructures revealed that larger numbers of GBF, SPF, and AF were produced, and their sizes became smaller with increasing ultrasonic power. As shown in Figure 10f, the width of GBF decreased from 31.6 to 8.1 μm, with increasing ultrasonic power to 1200 W. 

Some researchers reported that melt flow could be compared to the classic hydrodynamic problem that flows past a cylinder [32]. They believed that melt flow was the turbulence with rapid heat transfer. Stronger turbulence can more easily break the dendrites [14]. The Reynolds number (Re) can be used to estimate the intensity of turbulence, as defined in Equation (2).

(2) $$Re=\frac{\rho vL}{\mu }$$

where ρ is the melt density, v is the mean velocity of melt flow, L is the characteristic length, and μ is the melt dynamic viscosity. The characteristic length could be approximately the value of weld width. The value of Re was proportional to the values of L and v. The melt flow improved by ultrasonic vibration increased the weld width and melt flow velocity. In previous studies, the calculated value of Re increased from 5614 to 11389, with the ultrasonic power increased from 0 to 60% [33]. Ultrasonic vibration accelerated the melt flow, which broke the stable status of grain growth. As a result, the number and proportion of PF were significantly decreased. In addition, many dendrite fragments broken by ultrasonic cavitation induced new nucleation. This result might account for the microstructure refinement and increased amounts of GBF.

**Figure 11:**
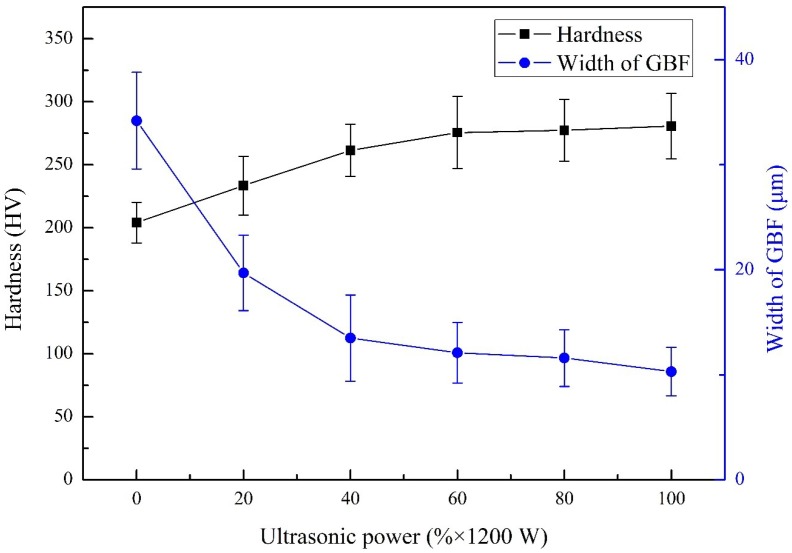
The average hardness of weld metal and width of grain boundary ferrite (GBF) in the deposited metal of joints welded at different ultrasonic output power.

Figure 11 shows the average hardness of weld metal and the width of GBF microstructures obtained at different ultrasonic power. The variation trend of hardness was contrary to the width of grain boundary ferrite. The hardness of the deposited metal welded without ultrasonic-assisted was 204 HV because of the high content of PF and large grains. As the ultrasonic power increased to 20% and 60%, the average hardness was 234 HV and 276 HV. The hardness values were increased by 14.4% and 35%, respectively, compared to those of the deposited metal welded without ultrasonic-assisted. The hardness improvement of the deposited metal welded with ultrasonic-assisted could be explained by two primary factors. One factor was the evolution of microstructure in the deposited metal. The proportion of PF with lower hardness decreased, and the contents of harder microstructure increased, such as GBF and SPF. Another factor was the microstructure refinement. For instance, the average width of GBF exhibited a decrease of 70%, from 34.2 to 10.3 μm, with increasing ultrasonic power to 100% of maximum output power. With the ultrasonic power increased continuously to 100%, the hardness increased to 281 HV. Compared to that of weld obtained at 60% ultrasonic power, this hardness value only increased by about 15 HV, which indicated that the promoting effect on microstructure refinement caused by ultrasound was limited at a higher level of ultrasonic power.

### 3.4. Mechanical Properties

**Figure 12:**
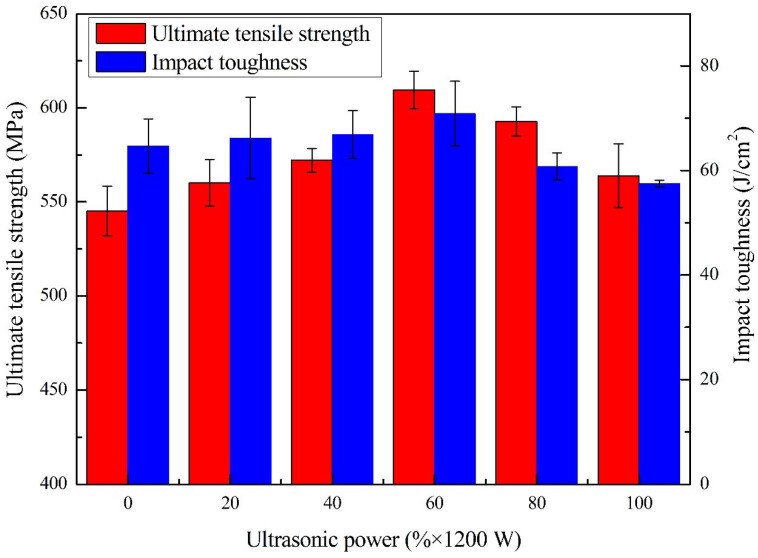
Ultimate tensile strength and impact toughness of the weld joints welded at different ultrasonic output power.

Table 2 and Figure 12 exhibit the ultimate tensile strength and impact toughness of the joints welded at different ultrasonic output power. The results exhibited that both of them firstly increased and then decreased with the increase of ultrasonic power. In the general welded joint without ultrasonic, the tensile strength and impact toughness were 545 MPa and 65 J/mm^2^, respectively. With the introduction of ultrasonic energy, there were significant increases in both tensile strength and impact toughness of the welded joint. The maximum tensile strength of 610 MPa and impact toughness of 71 J/mm^2^ were obtained in the joint welded with ultrasonic-assisted at 60% of maximum output power, which increased by 11.8% and 9.6%, respectively. Then, both tensile strength and impact toughness decreased when ultrasonic power continued to increase. The minimum tensile strength of 564 MPa and impact toughness of 58 J/mm^2^ were obtained in the joints welded at 100% of maximum ultrasonic power, respectively. According to the study in chapter 3.3, there is reason to believe that the various laws of tensile strength and impact toughness of joints have a close relationship with the microstructure and hardness of the deposited metal.

**Figure 13:**
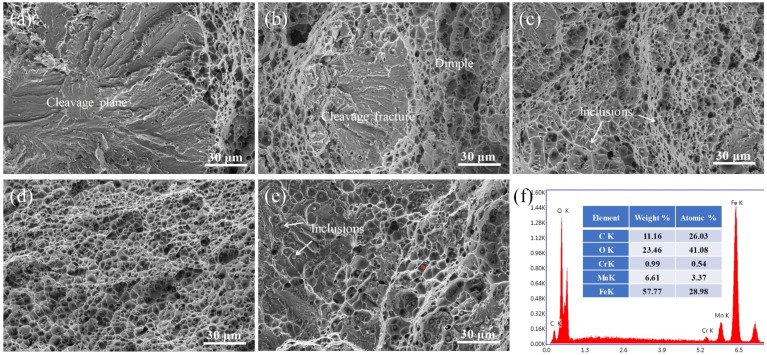
Tensile fracture morphology of joints welded at different ultrasonic output power: (**a**) without ultrasonic, (**b**) 20%, (**c**) 40%, (**d**) 60%, (**e**) 80%, and (**f**) EDS results for the inclusion marked in Figure 13e.

In the tensile strength test, almost all fractures initiated at HAZ, and then further propagated along defects until the samples fracture. A number of studies have suggested that the brittle martensite and the high stresses formed in the HAZ during rapid cooling are the major reasons resulting in the crack initiation [8,9,10]. Figure 13 shows the typical fracture morphology of joints welded under different ultrasonic power. As shown in Figure 13a, without ultrasonic-assisted, the fracture modes were typical cleavage fracture because the fracture occurred at hydrogen-induced cracks and then extended to the weld metal. With increasing ultrasonic power, the area of the cleavage plane was decreased because the finer microstructure inhibited the propagation of microcrack, as shown in Figure 13b. Figure 13c–e show the typical ductile fracture, and the fracture surface was full of dimples. Compared with others, Figure 13d shows that the fracture exhibited deeper and more evenly distributed dimples, which was consistent with the highest tensile strength of the joint welded at 60% ultrasonic power.

Energy-dispersive spectroscopy (EDS) result showed that the inclusion marked by a red cross symbol was consisted of C, O, Fe, Mn, and trace amounts of Cr, as shown in Figure 13f. This indicated that FeO and MnO were the primary components of inclusions. The large amounts of inclusions might be caused by the rapid solidification of weld metal.

**Figure 14:**
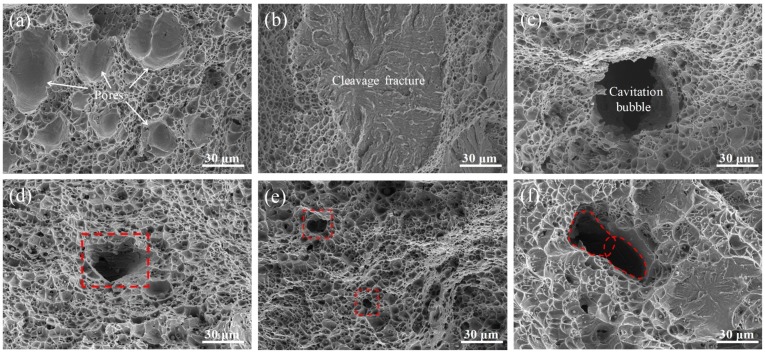
Impact fracture morphology of joints welded at different ultrasonic output power: (**a**) and (**b**) without ultrasonic-assisted, (**c**) 20%, (**d**) 40%, (**e**) 60%, and (**f**) 100%.

Figure 14 shows the impact fracture morphology of joints at different ultrasonic power. As shown in Figure 14a, some pores were produced on the ductile fracture surface of joint welded without ultrasonic. In general, the diameters of these pores were 20–50 μm, and they were relatively shallow and closely packed. Besides the ductile fracture, there were some local cleavage fractures that occurred in the impact test, as shown in Figure 14b. It was worth noting that there was a small number of deep holes distributed in the weld metal, as shown in Figure 14c. Some reports have indicated that ultrasonic cavitation can break the large bubble into several smaller bubbles [34]. So, this deep hole perhaps was the trace left by the broken bubble that could not escape from the molten pool before solidification. Figure 14c–e show that the size of the hole significantly decreased from about 50 μm to 10 μm with the increase of ultrasonic. It could be inferred that cavitation bubbles caused by ultrasonic remained these smaller holes in the deposited metal. As shown in Figure 14f, the area marked by the red dotted line indicated that two cavitation bubbles coalesced to a rod-like bubble and remained in the solidified metal. The pores induced by many cavitation bubbles and some cleavage fracture appearances proved the decrease of impact toughness of joint welded at a relatively high ultrasonic output power.

The cavitation bubbles that could escape from the weld pool had a critical size. The value of this size could be estimated as the following formula:

(3) $$v_{e}=\frac{2\left(\rho_{L}-\rho_{G}\right)gR^{2}}{9\eta }$$

where v_e_ is the velocity of escaping from the weld pool, ρ_L_ and ρ_G_ are the density of the molten steel and the density of the gas in bubbles, respectively, g is the gravity constant, η is the viscosity of the melt, and R the radius of the cavitation bubble. The value of the v_e_ could be calculated by dividing weld penetration depth (P) by solidification time (t) of the weld pool. In the ordinary welding without ultrasonic (P and t were 2.36 mm and 6.7 s, respectively), the minimal escaping diameter for the cavitation bubble was approximately 22 µm [33]. It meant that cavitation bubbles would be left in the weld pool and became pores in the case of that the cavitation bubble was smaller than the critical value of 22 µm. Furthermore, ultrasonic vibration enhanced the melt flow during the welding process, which induced the deepening in the weld penetration. The enhanced melt flow and slag removal caused by ultrasonic also perhaps decreased the solidification time of the weld pool. So, in effect, the critical diameter of the bubble was more than 22 µm during the ultrasonic-assisted welding process. It meant that more cavitation bubble was left in the weld metal, which might be one reason that weld mechanical property decreased when the ultrasonic output power was further increased.

## 4. Conclusions

Ultrasonic vibration enhanced the melt flow and improved the weld appearance to some extent. However, a high-level ultrasonic power would break the slag covered on the weld and result in the generation of more welding spatters.

The width of grain boundary ferrite (GBF) decreased from 38 to 12 μm, and the hardness increased from 204 to 276 HV as the ultrasonic power decreased to 1200 W. The tensile strength of the joint increased from 545 to 610 MPa, and the impact toughness increased from 65 to 71 J/mm^2^ when the power increased to 60%; that is, the ultrasonic power value of about 700 W was the most beneficial for the mechanical properties of welded joints.

The cavitation bubble induced by ultrasonic would be left in the weld metal and became welding pores. The number of these pores increased with increasing ultrasonic power, which might be one reason why the mechanical property of weld decreased at a relatively high ultrasonic output power.

## Figures and Tables

**Table 1:** Chemical composition of E40 and H08A steel.

Material	C	Mn	Ni	Cr	Si	P	S	Fe
E40	0.17	1.35	0.04	0.01	0.46	0.005	0.30	Bal.
H08A	0.10	0.40	0.01	0.01	0.05	0.025	0.025	Bal.

**Table 2:** Mechanical properties of joints.

Ultrasonic Power (% × 1200W)	0	20	40	60	80	100
Ultimate strength (MPa)	545	560	572	610	593	564
Fracture location	Welds	Welds	Welds	BM	Welds	Welds
Impact toughness (J/cm^2^)	65	66	67	71	61	58

## Supplementary Materials

The following are available online at https://www.mdpi.com/1996-1944/13/6/1442/s1, Video S1: Melt flow and droplet transfer process during conventional wet welding process without ultrasonic under water. (The playback speed is 20 times slower than welding process). Video S2: Melt flow and droplet transfer process during ultrasonic-assisted wet welding process when the ultrasonic output power was 40%. (The playback speed is 20 times slower than welding process.). Video S3: Melt flow and droplet transfer process during ultrasonic-assisted wet welding process when the ultrasonic output power was 80%. (The playback speed is 20 times slower than welding process.)
