# Supplementary figures and images for: Investigating the Advantages of Ultrasonic-assisted Welding Technique Applied in Underwater Wet Welding by in-situ X-ray Imaging Method
Source: Materials (Basel). 2020 Mar 21;13(6):1442. doi: 10.3390/ma13061442 (PMC7142934; doi:10.3390/ma13061442)

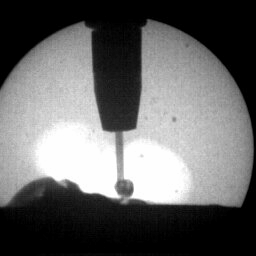

Supplement: Supplementary file 1 [file materials-13-01442-s001.zip › Supplementary materials/Supplementary Movie 1.gif]

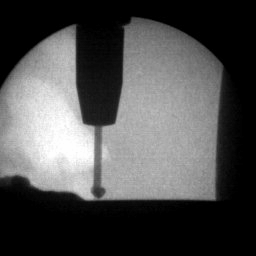

Supplement: Supplementary file 1 [file materials-13-01442-s001.zip › Supplementary materials/Supplementary Movie 2.gif]

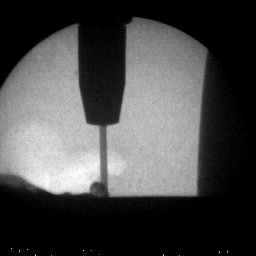

Supplement: Supplementary file 1 [file materials-13-01442-s001.zip › Supplementary materials/Supplementary Movie 3.gif]
